# Exploring the impact of varying definitions of exacerbations of chronic obstructive pulmonary disease in routinely collected electronic medical records

**DOI:** 10.1371/journal.pone.0292876

**Published:** 2023-11-01

**Authors:** Hannah Whittaker, Kieran J. Rothnie, Jennifer K. Quint

**Affiliations:** 1 School of Public Health & National Heart and Lung Institute, Imperial College London, London, United Kingdom; 2 Epidemiology, Value Evidence and Outcomes, Global Medical R&D, GSK, London, United Kingdom; University of Oxford, UNITED KINGDOM

## Abstract

**Background:**

Validity of exposure and outcome measures in electronic medical records is vital to ensure robust, comparable study findings however, despite validation studies, definitions of variables used often differ. Using exacerbations of chronic obstructive pulmonary disease (COPD) as an example, we investigated the impact of potential misclassification of different definitions commonly used in publications on study findings.

**Methods:**

A retrospective cohort study was performed. English primary care data from the Clinical Practice Research Datalink Aurum database with linked secondary care data were used to define a population of COPD patients ≥40 years old registered at a general practice. Index date was the date eligibility criteria were met and end of follow-up was 30/12/19, death or end of data collection. Exacerbations were defined using 6 algorithms based on definitions commonly used in the literature, including one validated definition. For each algorithm, the proportion of frequent exacerbators (≥2 exacerbations/year) and exacerbation rates were described. Cox proportional hazard regression was used to investigate each algorithm on the association between heart failure and risk of COPD exacerbation.

**Findings:**

A total of 315,184 patients were included. Baseline proportion of frequent exacerbators varied from 2.7% to 15.3% depending on the algorithm. Rates of exacerbations over follow-up varied from 19.3 to 66.6 events/100 person-years. The adjusted hazard ratio for the association between heart failure and exacerbation varied from 1.45, 95% confidence intervals 1.42–1.49, to 1.01, 0.98–1.04.

**Interpretation:**

The use of high validity definitions and standardisation of definitions in electronic medical records is crucial to generating high quality, robust evidence.

## Introduction

Electronic medical records (EMR) consist of data routinely collected as part of clinical care and are commonly used for health care research. They have many strengths including large population sizes, a wide breadth of health-related information and are often more generalisable to wider populations, making EMR databases ideal to investigate routine clinical practice. One important aspect of EMR data quality is validity of variables of interest, such as study exposures and outcomes. Poor quality definitions can lead to identification of incorrect variables and misclassification of events, leading to varying study results including over or underestimation of disease prevalence and limited generalisability of study findings [1]. Validation of study exposure and outcome definitions is essential to ensure robust, comparable study findings.

Validation studies of different EMR databases are used to develop algorithms with high accuracy in identifying people with specific diseases. These have reported high positive predictive values (PPV) and sensitivity across a number of diseases, including chronic obstructive pulmonary disease (COPD), asthma, and cardiovascular diseases which indicate how likely a recorded disease diagnosis is true [2–5]. Studies have also validated disease-specific events such as exacerbations of COPD, as people with COPD often experience exacerbations, which are widely studied in EMR [6–11].

Prospective studies, including randomized controlled trials, have clear definitions of study endpoints that are comparable between studies. Studies using EMR do not always have clear clinical definitions, especially for exacerbations of COPD that can be defined in more than one way. The use of standardized or validated algorithms are recommended across study endpoints and should be used to ascertain exacerbations of COPD using EMR [12]. Multiple definitions have been used over the years to define exacerbations of COPD, including several definitions known to have poorer accuracy in identifying events [13–15]. Additionally, reporting is poor, and it is not always clear exactly how exacerbation events have been defined. Whether the use of multiple definitions commonly in use lead to significant misclassification and different study findings has not been explored.

We investigated the potential impact of misclassification of exacerbations of COPD on study findings using a selection of definitions commonly used in the published literature. We aimed to investigate the impact of different definitions where COPD exacerbations are used as incident events, prevalent events, as an exposure, and as an outcome.

## Methods

### Study population

We used Clinical Practice Research Datalink (CPRD) Aurum, a primary care routinely collected database. Linked Hospital Episode Statistics (HES) admitted patient care (APC), a secondary care routinely collected database, was provided for this study by CPRD for patients in England. All data were pseudonymised. CPRD Aurum consists of general practices (GPs) from England and is representative of the English population in terms of geographic location, age, sex, and socioeconomic deprivation [16]. Using CPRD Aurum, we defined a population of people diagnosed with chronic obstructive pulmonary disease (COPD) who were over the age of 40 years old and were current or ex-smokers. A COPD diagnosis was determined using a primary care COPD diagnosis code alone following a previous validation study of COPD patients in CPRD [2]. COPD patients were included if they were eligible for linkage with HES, had data recorded in CPRD Aurum from 1^st^ January 2010 onwards, had at least one year of data recorded from the time they registered with the GP, and had at least one GP consultation recorded in CPRD Aurum prior to index date. Patient’s index date was defined as the date at which all criteria were satisfied. End of follow-up was defined as the 30^th^ of December 2019, or earlier if patients died or left the GP practice.

### Exacerbations of COPD

Six algorithms for defining exacerbations of COPD were chosen based on common definitions of exacerbations of COPD, including a validated definition with known high accuracy when compared to patient notes. CPRD Aurum was used for recorded primary care events and HES for events requiring hospitalisation.

Algorithm 1: Based on a validated algorithm for identifying exacerbations of COPD that had been developed using CPRD [6,7]. Exacerbation events were defined by a code for an exacerbation or a lower respiratory tract infection (LRTI) in primary care, or codes for 2/3 of chronic cough, breathlessness, and/or sputum recorded on the same day as a prescription of respiratory-related antibiotics and oral corticosteroids prescribed for a duration of 5–14 days (https://github.com/NHLI-Respiratory-Epi/AECOPD). Hospitalised exacerbation events were defined as an ICD10 code recorded in HES (ICD10 J44.1 in any position, J44.0 in any position, or J44.9 in the first position). Exacerbation events that were recorded within 14 days of one another and on the same day as a COPD annual review visit were excluded. This validated algorithm provides a positive predictive value of 86% and sensitivity of 63% for GP recorded exacerbations and a sensitivity of 87.5% for hospitalised exacerbations [6,7].

Algorithm 2: A subset of algorithm 1 for defining primary care events alone [6,17]. HES was not used to determine events. Exacerbation events that were recorded within 14 days of one another and on the same day as a COPD annual review visit were excluded.

Algorithm 3: Based on previous studies that used prescription data in combination with codes for LRTIs to identify moderate exacerbation events [18]. This included a code for a LRTI recorded on the same day as a prescription for an oral corticosteroid or on the same day as a respiratory-related antibiotic, or on the same day as both an oral corticosteroid and a respiratory-related antibiotic.

Algorithm 4: Use of exacerbation codes only recorded in primary care. No other criteria were applied [19–21].

Algorithm 5: Based on studies that only used oral corticosteroid prescriptions to determine exacerbation events in primary care [15,22]. Exacerbation events were defined as having an oral corticosteroid prescription in primary care for a duration of 5–14 days.

Algorithm 6: Aimed at exploring whether the addition of HES accident and emergency (A&E) data in combination with HES APC helped to determine additional exacerbation events. Specifically, exacerbation events were defined in the same way as for definition 1 with the addition of HES A&E events. These events were defined as having an A&E diagnosis code for a non-asthma respiratory cause. In addition, exacerbation events that were recorded within 14 days of one another and on the same day as a COPD annual review visit were excluded.

For each definition, exacerbation events were identified in the year prior to index date as well as over the follow-up period. Patients were categorised as frequent exacerbators if they had 2 or more exacerbation events recorded in a single year.

### Statistical analyses

The following analyses were chosen where COPD exacerbations were used as incident events, prevalent events, as an exposure, and as an outcome to investigate the impact of varying definitions of exacerbations of COPD. For each definition, we described the proportion of baseline frequent exacerbators and patient demographics.

We described the proportion of frequent exacerbators for each year of patient follow-up using each exacerbation definition. Rates of exacerbations during follow-up and by calendar year (2010 to 2019) were calculated by dividing the total number of events over follow-up, or for each calendar year, by the total contributing person-time over follow-up, for each calendar year. We described the rates of exacerbations over follow-up excluding exacerbations events that were within 2 weeks of one another or events that were on the same date as a COPD annual review visit. These exclusion criteria were part of algorithms one, two, and six, but not compulsory for three, four and five. Previous studies using these algorithms did not always exclude events based on proximity to the subsequent exacerbation or based on annual review visit. To better contextualise and compare rates of exacerbations between algorithms, we estimated rates that included and excluded these events to keep conditions as similar as possible between algorithms other than the codes used.

Third, we used Cox proportional hazard regression to estimate the association between baseline heart failure and risk of first exacerbation, an established association, over follow-up using each of the six definitions [23]. Baseline heart failure was defined as a diagnosis recorded in CPRD Aurum. Models were adjusted for age, sex, and smoking status.

### Sensitivity analysis

To test for misclassification of A&E codes related to algorithm six, we determined the proportion of patients who had at least 1 A&E non-asthma respiratory code and no corresponding admission code and the proportion of patients who had at least 1 A&E non-asthma or bronchial asthma code and no corresponding admission code.

### Exploratory analysis

CPRD have two primary care EMR databases that collected the same data using two different software programmes: Vision and EMIS. EMIS is used to collect data at GPs to form the CPRD Aurum database which contains information from GPs in England only [16]. Vision is used to collect data at GPs to form the CPRD GOLD database which contains information from GPs in England, Scotland, and Wales [24]. To investigate differences in CPRD databases, we used CPRD GOLD to define exacerbations the same way as algorithm one in CPRD Aurum. We described proportions of frequent exacerbators at baseline and during follow-up, estimated exacerbation event rates over follow-up, and investigated the association between baseline heart failure and risk of first exacerbation during follow-up and compared estimates to those reported using CPRD Aurum.

### Ethical approval

The protocol for this research was approved by the Independent Scientific Advisory Committee (ISAC) for MHRA Database Research (protocol number 21001666) and the approved protocol was made available to the journal and reviewers during peer review. This study is based in part on data from the Clinical Practice Research Datalink obtained under licence from the UK Medicines and Healthcare products Regulatory Agency. The data is provided by patients and collected by the NHS as part of their care and support. The interpretation and conclusions contained in this study are those of the author/s alone. Linked pseudonymised data was provided for this study by CPRD. Data is linked by NHS Digital, the statutory trusted third party for linking data, using identifiable data held only by NHS Digital. Select general practices consent to this process at a practice level with individual patients having the right to opt-out.

This study is based in part on data from the Clinical Practice Research Datalink (CPRD) obtained under licence from the UK Medicines and Healthcare products Regulatory Agency. The data is provided by patients and collected by the National Health Service (NHS) as part of their care and support. The Office for National Statistics (ONS) was the provider of the ONS Data contained within the CPRD Data and maintains a Copyright © 2019, re-used with the permission of The Health & Social Care Information Centre, all rights reserved. The interpretation and conclusions contained in this study are those of the authors alone.

## Results

A total of 315,184 COPD patients were included in the analysis (**S1 Fig**). Using algorithm one, 46,555 (14.8%) patients were defined as frequent exacerbators at baseline. However, a total of 41,247 (13.1%), 21,724 (6.9%), 8,480 (2.7%), 13,326 (4.2%), and 48,361 (15.3%) of included COPD patients were defined as frequent exacerbators at baseline following algorithms two, three, four, five, and six, respectively. Patients defined as frequent exacerbators were similar in terms of age and socioeconomic deprivation, however patients defined as frequent exacerbators using algorithm five included a lower proportion of men and current smokers compared with frequent exacerbators using all other algorithms (**Table 1**).

**Table 1:** Baseline characteristics of COPD patients defined as frequent exacerbators at baseline using six different algorithms to define exacerbations of COPD.

	Algorithm 1	Algorithm 2	Algorithm 3	Algorithm 4	Algorithm 5	Algorithm 6
**Mean age (SD)**	68.4 (0.05)	68.1 (0.06)	67.9 (0.08)	69.4 (0.12)	67.6 (1.00)	68.4 (0.05)
**Male sex**	21,867 (47.0)	19,317 (46.8)	10,026 (46.2)	4,062 (47.9)	5,849 (43.9)	22,840 (47.2)
**Current smoking**	21,652 (46.5)	19,309 (46.8)	10,224 (47.1)	3,739 (44.1)	5,852 (43.9)	22,545 (46.6)
Region* Northeast Northwest Yorkshire E Midlands W Midlands East England London Southeast Southwest	2,661 (5.7) 13,300 (28.6) 2,090 (4.5) 953 (4.5) 8,228 (17.7) 1,821 (3.9) 4,682 (10.1) 7,411 (15.9) 5,394 (11.6)	2,391 (5.8) 11,942 (29.0) 1,876 (4.6) 844 (2.1) 7,362 (17.9) 1,634 (4.0) 3,902 (9.5) 6,499 (15.8) 4,782 (11.6)	1,136 (5.2) 6,526 (30.1) 990 (4.6) 476 (2.2) 3,999 (18.4) 883 (4.1) 1,782 (8.2) 3,477 (16.0) 2,448 (11.3)	579 (6.8) 2,739 (32.3) 373 (4.4) 162 (1.9) 1,385 (16.3) 260 (3.1) 597 (7.0) 1,106 (13.1) 1,275 (15.0)	1,088 (8.2) 3,716 (27.9) 525 (3.9) 321 (2.4) 2,329 (17.5) 476 (3.6) 1,201 (9.0) 2,131 (16.0) 1,528 (11.5)	2,843 (5.9) 13,750 (28.4) 2,165 (4.5) 978 (2.0) 8,558 (17.8) 1,858 (3.8) 4,929 (10.2) 7,714 (16.0) 5,524 (11.4)
**IMD** 1 (most deprived) 2 3 4 5 (least deprived)	5,671 (12.2) 7,554 (16.2) 8,305 (17.9) 10,221 (22.0) 14,780 (31.8)	5,070 (12.3) 6,724 (16.3) 7,367 (17.9) 9,038 (21.9) 13,025 (31.6)	2,681 (12.4) 3,634 (16.7) 3,817 (17.6) 4,655 (21.4) 6,929 (31.9)	986 (11.6) 1,364 (16.1) 1,498 (17.7) 1,872 (22.1) 2,753 (32.5)	1,564 (11.8) 2,120 (15.9) 2,382 (17.9) 2,942 (22.1) 4,307 (32.4)	5,849 (12.1) 7,813 (16.2) 8,623 (17.8) 10,624 (22.0) 15,425 (31.9)

Legend: Algorithm 1 was based on a validated algorithm using CPRD and HES, algorithm 2 included the validated CPRD algorithm but no HES, algorithm 3 included lower respiratory tract infection and prescribed medications for exacerbations, algorithm 4 included exacerbation of COPD codes in CPRD alone, algorithm 5 included oral corticosteroid prescriptions alone, and algorithm 6 included the validated CPRD and HES algorithm as well as accident and emergency HES data. COPD (chronic obstructive pulmonary disease). *Approximately 0.03% of all frequent exacerbators had missing Region.

### Frequency and rate of exacerbations over follow-up

**Fig 1:**
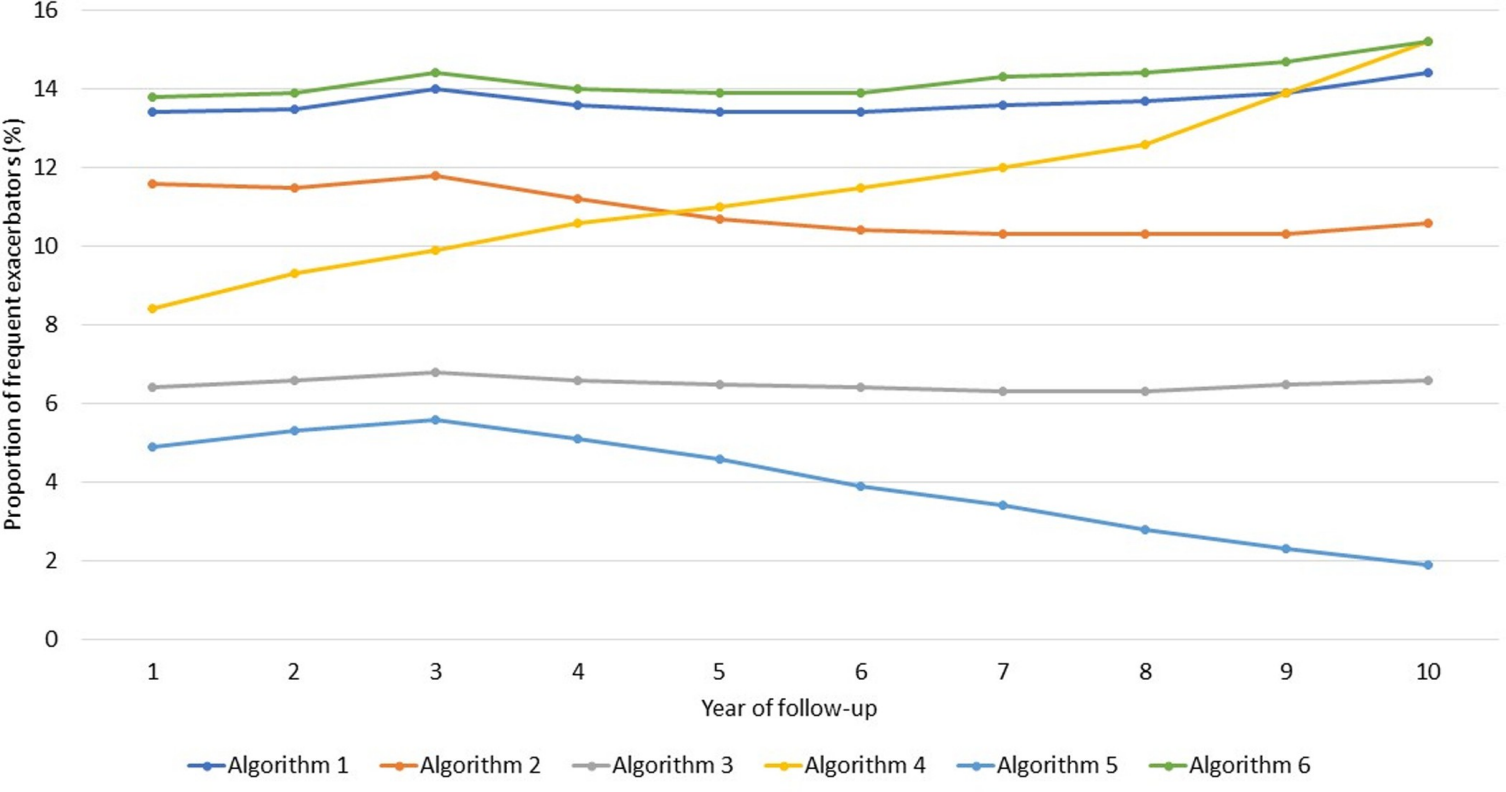
Proportion of frequent exacerbators for each year of follow-up. Legend: Numbers reported are percentages of the total population. The proportion of frequent exacerbators each year of follow-up was determined for patients with active follow-up for each year of interest. Algorithm 1 was based off a validated algorithm using CPRD and HES, algorithm 2 included the validated CPRD algorithm but no HES, algorithm 3 included lower respiratory tract infection and prescribed medications for exacerbations, algorithm 4 included exacerbation of COPD codes in CPRD alone, algorithm 5 included oral corticosteroid prescriptions alone, and algorithm 6 included the validated CPRD and HES algorithm as well as accident and emergency HES data.

The proportion of frequent exacerbators defined using algorithms one and six (i.e., using the validated CPRD, HES algorithm, and HES accident and emergency data) were relatively stable over follow-up and very similar (14.4% vs. 15.2% of total COPD population in year 10 of patient follow-up) (**Fig 1**). The proportion of frequent exacerbators defined using algorithm two (i.e., the validated CPRD algorithm alone) were lower than those reported using algorithms one and six and remained relatively stable over follow-up (from 11.6% to 10.6% in year 1 and 10, respectively). The proportion of frequent exacerbators defined using algorithm three (i.e., using LRTI and prescriptions) was much lower but remained stable over patient follow-up (from 6.4% to 6.6% in year one and ten, respectively). However, the proportion of frequent exacerbators defined using algorithm four (i.e., exacerbation codes) increased from 8.4% in year one of patient follow-up to 15.2% in the tenth year of patient follow-up. The lowest proportion of frequent exacerbators was seen when using algorithm five (i.e., OCS prescriptions) and the proportion declined from 4.9% in year 1 to 1.9% in year 10.

Exacerbation rates were highest for events defined using algorithms one, four and six (**Table 2**). The lowest rate of exacerbations was seen for algorithm five. However, once events within 2 weeks of one another and events recorded on the same day as a COPD annual review were excluded, rates of exacerbations for algorithms three, four, and five were lower.

**Table 2:** Mean rates of exacerbations over follow-up.

	Exacerbation rates per 100PY follow-up	
Exacerbation definition	No exclusion	Full exclusion
Algorithm 1	64.4 (64.3–64.5)	64.4 (64.3–64.5)
Algorithm 2	54.1 (54.0–54.2)	54.1 (54.0–54.2)
Algorithm 3	35.2 (35.1–35.3)	30.9 (30.8–31.0)
Algorithm 4	64.4 (64.2–64.5)	21.9 (21.8–21.9)
Algorithm 5	26.0 (25.9–26.1)	19.3 (19.3–19.4)
Algorithm 6	66.6 (66.4–66.7)	66.6 (66.4–66.7)

Legend: Full exclusion criteria included removing exacerbation events that were within two weeks of one another and events recorded on the same date as a COPD annual review visit. Algorithm 1 was based on a validated algorithm using CPRD and HES, algorithm 2 included the validated CPRD algorithm but no HES, algorithm 3 included lower respiratory tract infection and prescribed medications for exacerbations, algorithm 4 included exacerbation of COPD codes in CPRD alone, algorithm 5 included oral corticosteroid prescriptions alone, and algorithm 6 included the validated CPRD and HES algorithm as well as accident and emergency HES data. PY (person-year).

**Fig 2:**
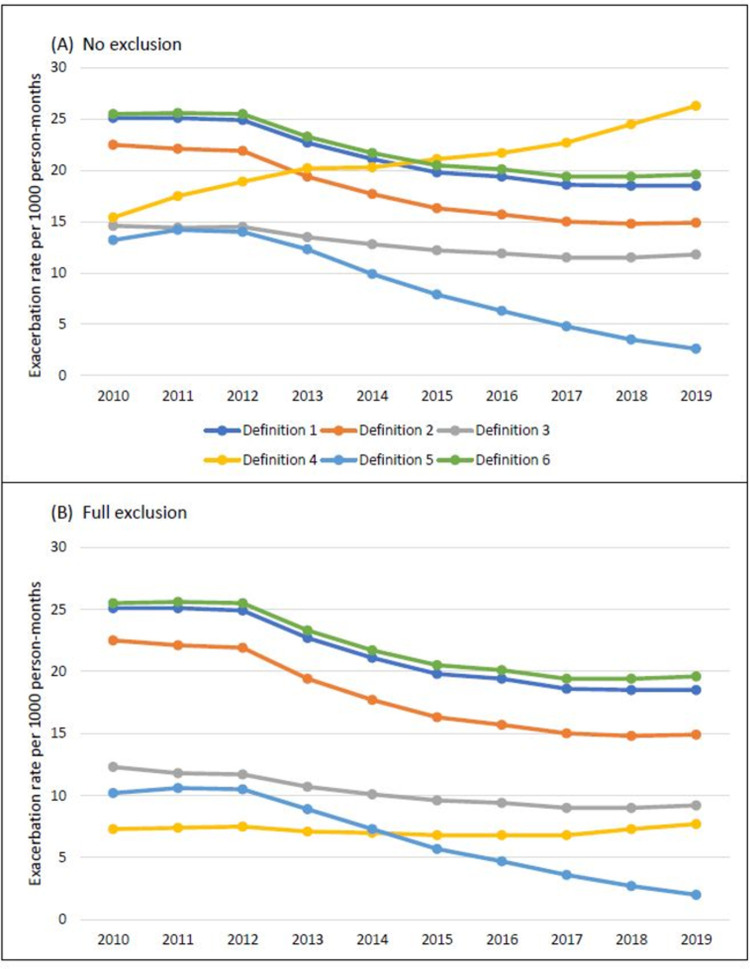
Calendar year exacerbation rates defined using A) six different algorithms and B) excluding exacerbation events that are within 2 weeks of one another or recorded on the same date as a COPD annual review visit. Legend: Rates are per 1000 person-months for each calendar year between 2010 and 2019 for patients with active follow-up in each specific year. Algorithm 1 was based off a validated algorithm using CPRD and HES, algorithm 2 included the validated CPRD algorithm but no HES, algorithm 3 included lower respiratory tract infection and prescribed medications for exacerbations, algorithm 4 included exacerbation of COPD codes in CPRD alone, algorithm 5 included oral corticosteroid prescriptions alone, and algorithm 6 included the validated CPRD and HES algorithm as well as accident and emergency HES data.

Exacerbation rates per calendar year were higher using algorithms one, two, and six however, the rates declined between 2013 and 2016 (**Fig 2**). This trend was also seen using algorithm five and to a lesser degree algorithm three. The rates of exacerbations using these algorithms were much lower than those defined using algorithms one, two, and six. The yearly exacerbation rate defined using algorithm four increased from 2010 to 2019 in panel A however, after excluding events that were within two weeks of one another and events that were recorded on the same date as a COPD annual review visit, the yearly exacerbation rate remained stable and at a lower rate.

### Association between heart failure and risk of exacerbations of COPD

**Fig 3:**
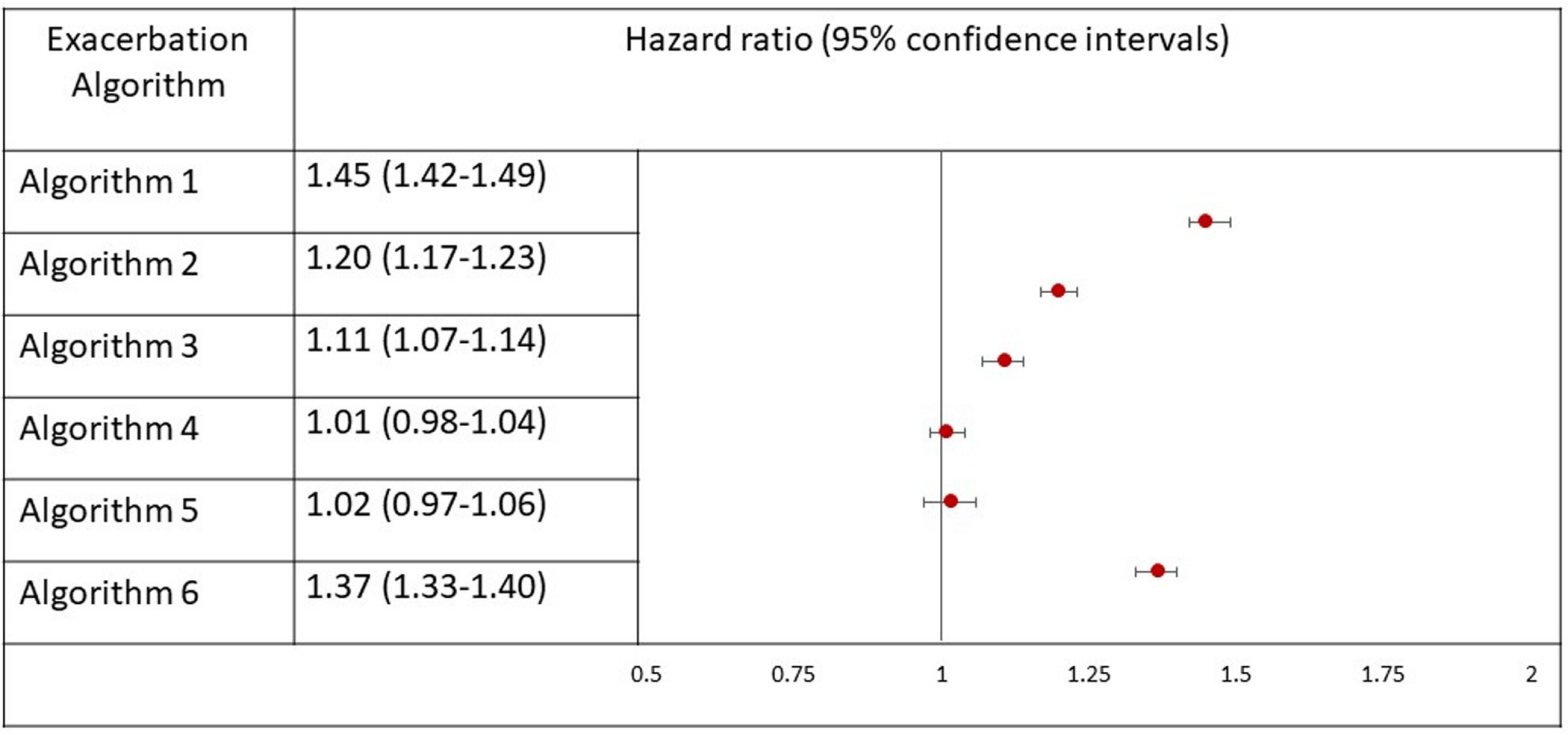
Association between baseline heart failure and risk of first exacerbation during follow-up in COPD patients using six different algorithms for defining exacerbation events. Legend: COPD (chronic obstructive pulmonary disease), HR (hazard ratio). Algorithm 1 was based off a validated algorithm using CPRD and HES], algorithm 2 included the validated CPRD algorithm but no HES, algorithm 3 included lower respiratory tract infection and prescribed medications for exacerbations, algorithm 4 included exacerbation of COPD codes in CPRD alone, algorithm 5 included oral corticosteroid prescriptions alone, and algorithm 6 included the validated CPRD and HES algorithm as well as accident and emergency HES data.

A significant association was seen between baseline heart failure and risk of future exacerbations using algorithm one (adjusted HR 1.46, 95% CI 1.43–1.50) (**Fig 3**). Results were similar when using algorithm six (adjusted HR 1.37, 95% CI 1.33–1.40). When using algorithm two and three, the magnitude of association was lower but still present (adjusted HR 1.20, 95% CI 1.17–1.23 and 1.11, 95% CI 1.07–1.14, respectively). The association was not significant when using algorithms four and five (adjusted HR 1.01 95% CI 0.98–1.04, and 1.02, 95% CI 0.97–1.06, respectively).

### Sensitivity analysis

At baseline, a total of 8,706 (2.8%) of COPD patients had at least one A&E event defined using a non-asthma respiratory code and no HES ACPC event. A total of 9,687 (3.1%) of COPD patients had at least one A&E event defined using a non-asthma respiratory code or a bronchial asthma code and no HES APC event.

### Exploratory analysis

A total of 263,636 COPD patients were included in CPRD GOLD, of which 37,429 (14.2%) were frequent exacerbators. Frequent exacerbators in CPRD GOLD were similar to COPD patients in CPRD Aurum in terms of age, sex, smoking status, and IMD however, CPRD GOLD frequent exacerbators varied in terms of practice region (**S1 Table**). The proportion of frequent exacerbators in the first year of follow-up was 15.6% and declined to 11.4% in the last year of patient follow-up (**S2 Table**). The proportion of frequent exacerbators in CPRD GOLD was higher for years one to six but lower for years six to ten compared with the proportion of frequent exacerbators in CPRD Aurum. The mean exacerbation rate over follow-up was 70.7 (95% CI, 70.6–70.9) per 100 person-years. This rate was slightly higher than that reported in CPRD Aurum (64.4, 95% CI 64.3–64.5). The annual exacerbation rate per calendar year of follow-up declined in a similar trend to that seen in CPRD Aurum (**S2 Fig**). Lastly, the adjusted hazard ratio for the association between baseline heart failure and risk of first exacerbation over follow-up was 1.21 (95% CI 1.17–1.26). This estimate was smaller than that reported in CPRD Aurum.

## Discussion

Using algorithms of lower validity to define exacerbations of COPD led to different study findings compared with our gold standard validated algorithm. We found the proportion of COPD patients categorised as frequent exacerbators differed at baseline and over follow-up as well as exacerbation rates over calendar year. We found the association between baseline heart failure and risk of exacerbations varied between algorithms, some algorithms resulting in no association. Our explorative work suggests that the use of secondary care admission data is sufficient in identifying hospitalised exacerbation events and most COPD patients who visit the emergency department for a respiratory-related reason are admitted to hospital. Overall, our findings show use of algorithms with poor validity and low accuracy can lead to incorrect study findings and validated algorithms should be used.

Definitions of exacerbations of COPD in CPRD have been validated previously [6,7]. The recommended algorithm with the highest validity included a combination of codes for exacerbations, LRTIs, oral corticosteroids, respiratory-related antibiotics, symptoms, and hospital exacerbation admissions. Criteria around the duration of a prescription, multiple events, and annual review visits in relation to recorded events was also recommended. This algorithm is available online (https://github.com/NHLI-Respiratory-Epi/AECOPD) and we encourage researchers to use it as we found a larger number of recorded exacerbation events were identified whereas other algorithms did not capture as many events and resulted in incorrect findings. Algorithms that excluded linked secondary care data resulted in the identification of fewer events. It is well known that severity of exacerbations of COPD is associated with varying risk of future outcome events such as cardiovascular disease and therefore not including hospitalised events could lead to an underestimation of risk by including fewer and less severe exacerbation events [9]. Algorithms that used LRTI codes alongside prescription codes missed events that could have been coded using exacerbation codes in both primary and secondary care. Algorithms that used prescription codes alone or exacerbations codes alone also led to misclassification of exacerbators resulting in a non-significant association with heart failure. For example, use of nonspecific antibiotic codes to define exacerbations of COPD has led to the identification of urinary tract infections rather than exacerbation events. Whist our findings highlight the consequences of using low validity definitions in EMRs, they also highlight the changes in coding practices by clinicians. Rates of exacerbations by calendar year show that primary care codes for exacerbations of COPD have increased from 2010 and in 2019. After excluding events that were on the same day as an annual review, the yearly rates were much lower suggesting exacerbation codes are being recorded much more frequently in recent years at an annual review visit. It is possible that the number of previous exacerbations could have been recorded in the free text to summarise the number of events a COPD patient experienced in that year. Using these codes alone with no other exclusion criteria could lead to overestimation of events [6]. Similarly, excluding events within two weeks of one another led to fewer exacerbation events highlighting the need to ensure duplicate events are taken into consideration as to not overestimate events.

### Previous literature

Previous studies have investigated the impact of using different definitions and data sources on disease outcomes. One study found the use of a combination of data sources including primary care, secondary care, death registration and disease registry data from the UK led to high positive predictive values of cancer. The use of primary or secondary care data alone resulted in much lower positive predictive value and sensitivity [25]. A study using EMRs from the Enterprise Data Warehouse found that the number of patients with atrial fibrillation varied by over 20% between five different definitions [26].

Studies that used algorithms with poor validity should be interpreted with caution. One study defined exacerbations of COPD using LRTI and prescription codes in primary secondary care data and categorised COPD patients into GOLD groups A-D. The association between GOLD group and lung function decline and future exacerbations of COPD were investigated [13,14]. This definition had a relatively high PPV and a low sensitivity, therefore, a smaller proportion of events were likely to be true exacerbation events. The Welsh Primary care Audit has previous used a single code to define exacerbations of COPD and found that only 11% of people with COPD exacerbated in a single year [19]. Similarly, a study found that 10% of people exacerbated within 30 days prior to index date when using oral corticosteroid codes alone to define exacerbations of COPD [15]. Studies using a validated definition of exacerbations found approximately 50% of people with COPD exacerbate in a single year [8,27] illustrating how the misuse of codes and definitions can lead to over and underestimation of events. The use of robust definitions and codes is essential to avoid inaccurate findings and bias. This has important implications for researchers using these data, for example when determining exacerbation frequency given its relationship with disease management and risk stratification. Our findings are also important for policy makers who rely on the data and illustrates the importance of standardising the use of these data and necessitating transparency in reporting definitions.

### Strengths and limitations

This is the first study to investigate how varying definitions of exacerbations of COPD used in electronic healthcare records can impact findings. We used data from CPRD Aurum, one of the most comprehensive electronic healthcare record databases in England. One limitation was use of data on the duration of respiratory-related antibiotics and oral corticosteroids to determine whether the duration was 5–14 days in length. This data had a high level of missingness in CPRD Aurum and could lead to fewer exacerbation events identified. Despite this, use of duration data would ensure high sensitivity of exacerbation events. The validated definition of exacerbations of COPD was validated in CPRD GOLD, a sister database of CPRD Aurum. We found some differences in study findings between the definition used in CPRD Aurum and the same definition in CPRD GOLD, which could be differences in geographic location as CPRD Aurum only covers England. Frequent exacerbators in CPRD Aurum also had slightly better socioeconomic status which could explain the slightly lower rates of exacerbations compared with CPRD GOLD. Despite this, differences were small, and our results showed that the use of the validated definition in CPRD Aurum remained superior to any of the other definitions used. Further studies could use quantitative bias analysis to quantify the differences in study findings between the definitions. In addition, one criterion that was applied to some algorithms excluded exacerbation events that were within two weeks of one another. Whilst this could lead to under-reporting of true exacerbation events that occurred less than two week of one another, this criterion has been previously used when validating exacerbations in CPRD data and given that the median length of time of an exacerbation of COPD is 14 days, the difference would be small [6,28].

## Conclusion

Use of different definitions of exacerbations of COPD with varying validity led to significant differences in study findings. In some cases, less valid definitions resulted in the reversal of study findings. Researchers must be comprehensive in the creation of case definitions in all EMR and claims databases in order to avoid biases. More importantly, validated definitions are essential to ensure accurate study findings to avoid over and underestimation of study events and misclassification. Not only are validated definitions within EMR databases crucial, but standardisation of definitions across EMR in different countries will generate high quality, robust and replicable real-world evidence across EMR research. Transparency in reporting study definition when publishing research findings is also crucial.

## Supporting information

S1 Checklist STROBE statement—checklist of items that should be included in reports of observational studies. (DOCX)

S1 Fig Flow diagram of patients included in the study. (PDF)

S2 Fig Calendar year exacerbation rates defined using algorithm 1 in CPRD Aurum and CPRD GOLD. Legend: Rates are per 1000 person-months for each calendar year between 2010 and 2019 for patients with active follow-up in each specific year. Algorithm 1 was based off a validated algorithm using CPRD and HES.
(PDF)

S1 Table Baseline characteristics between frequent exacerbators defined using algorithm 1 in CPRD Aurum and CPRD GOLD. Legend: SD (standard deviation), IMD (Index of Multiple Deprivation). * 22,401 (59.9%) of CPRD GOLD patients had missing data for IMD.
(PDF)

S2 Table Proportion of frequent exacerbators for each year of patient follow-up by algorithm used to define exacerbations of COPD. Legend: The proportion of frequent exacerbators each year of follow-up was determined for patients with active follow-up for each year of interest. Algorithm 1 was based off a validated algorithm using CPRD and HES, algorithm 2 included the validated CPRD algorithm but no HES, algorithm 3 included lower respiratory tract infection and prescribed medications for exacerbations, algorithm 4 included exacerbation of COPD codes in CPRD alone, algorithm 5 included oral corticosteroid prescriptions alone, and algorithm 6 included the validated CPRD and HES algorithm as well as accident and emergency HES data.
(PDF)
