# Supplementary figures and images for: Exploring the impact of varying definitions of exacerbations of chronic obstructive pulmonary disease in routinely collected electronic medical records
Source: PLoS One. 2023 Nov 1;18(11):e0292876. doi: 10.1371/journal.pone.0292876 (PMC10619826; doi:10.1371/journal.pone.0292876)

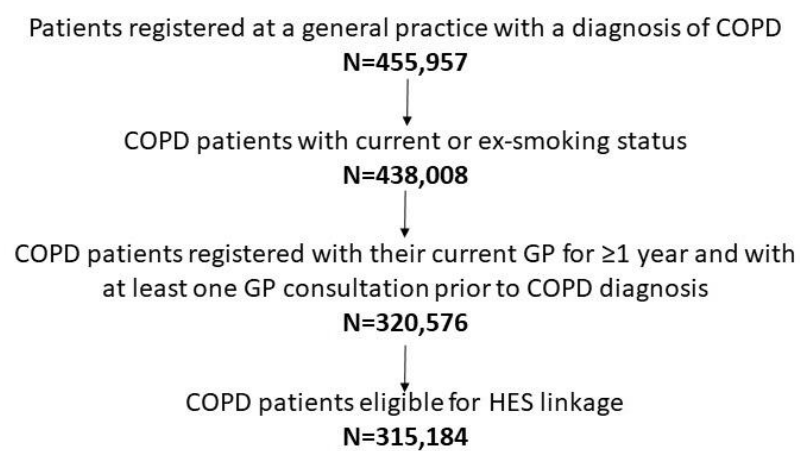

Figure S1: Flow diagram of patients included in the study.

Supplement: S1 Fig — (PDF) [file pone.0292876.s002.pdf]
