# Supplementary material for: Exploring the impact of varying definitions of exacerbations of chronic obstructive pulmonary disease in routinely collected electronic medical records
Source: PLoS One. 2023 Nov 1;18(11):e0292876. doi: 10.1371/journal.pone.0292876 (PMC10619826; doi:10.1371/journal.pone.0292876)
Supplement: S2 Fig — Legend: Rates are per 1000 person-months for each calendar year between 2010 and 2019 for patients with active follow-up in each specific year. Algorithm 1 was based off a validated algorithm using CPRD and HES. (PDF) [file pone.0292876.s003.pdf]

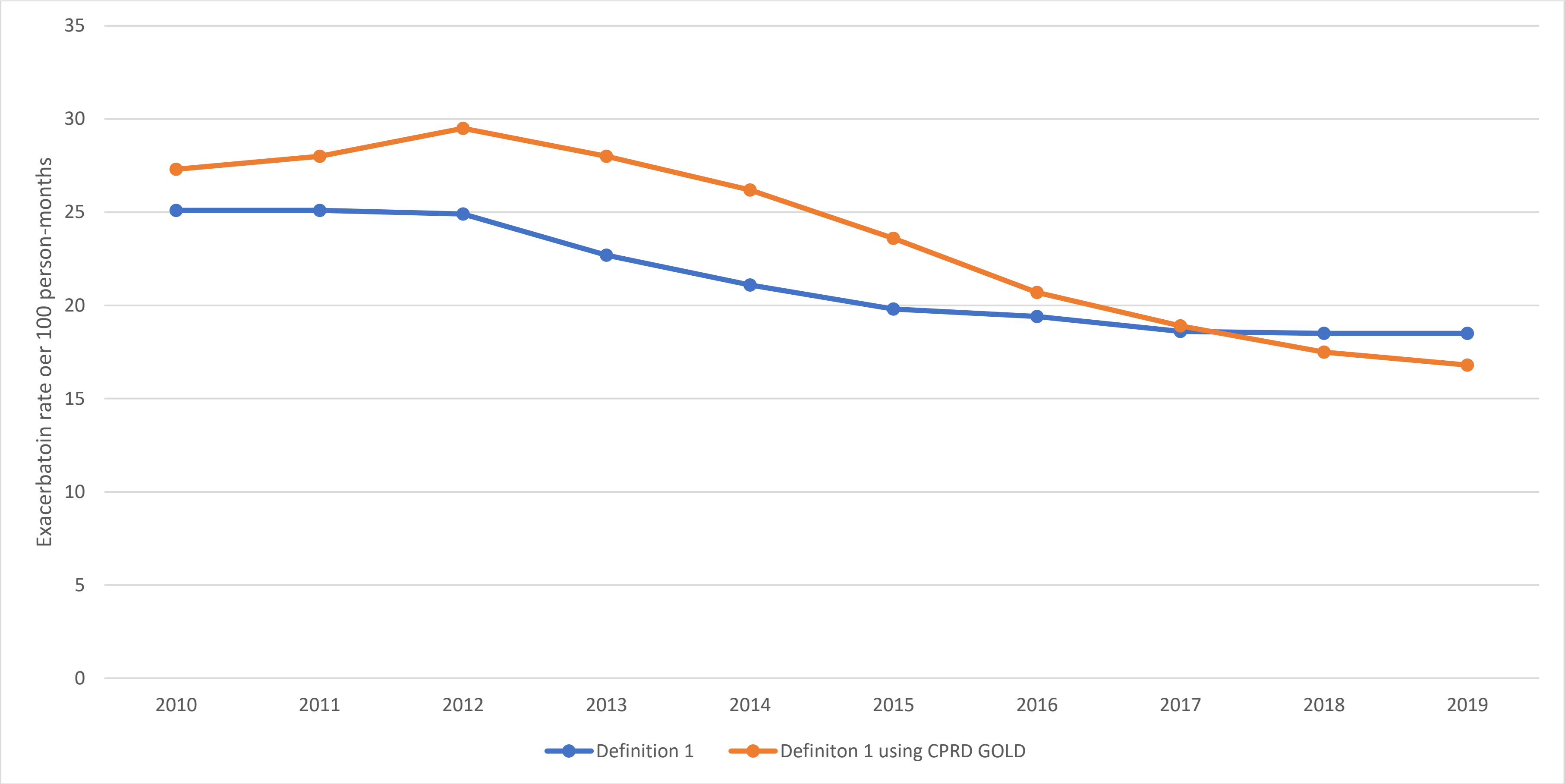

Figure S2: Calendar year exacerbation rates defined using algorithm 1 in CPRD Aurum and CPRD GOLD

Legend: Rates are per 1000 person-months for each calendar year between 2010 and 2019 for patients with active follow-up in each specific year. Algorithm 1 was based off a validated algorithm using CPRD and HES.
