# Supplementary material for: Exploring the impact of varying definitions of exacerbations of chronic obstructive pulmonary disease in routinely collected electronic medical records
Source: PLoS One. 2023 Nov 1;18(11):e0292876. doi: 10.1371/journal.pone.0292876 (PMC10619826; doi:10.1371/journal.pone.0292876)
Supplement: S1 Table — Legend: SD (standard deviation), IMD (Index of Multiple Deprivation). * 22,401 (59.9%) of CPRD GOLD patients had missing data for IMD. (PDF) [file pone.0292876.s004.pdf]

Table S1: Baseline characteristics between frequent exacerbators defined using algorithm 1 in CPRD Aurum and CPRD GOLD

	**Frequent exacerbators defined using algorithm 1**	
**Baseline characteristics**	**CPRD Aurum**	**CPRD GOLD**
**Mean age (SD)**	68.4 (11.4)	67.6 (11.4)
**Male sex**	21,867 (47.0)	16,673 (44.6)
**Current smoking**	21,652 (46.5)	15,684 (41.9)
**Region**		
Northeast	2,661 (5.7)	566 (1.5)
Northwest	13,300 (28.6)	4,704 (12.6)
Yorkshire	2,090 (4.5)	636 (1.7)
E Midlands	953 (4.5)	547 (1.5)
W Midlands	8,228 (17.7)	2,941 (7.9)
East England	1,821 (3.9)	1,560 (4.2)
London	4,682 (10.1)	2,492 (6.7)
Southeast	7,411 (15.9)	7,102 (19.0)
Southwest	5,394 (11.6)	2,316 (6.2)
South Central	-	4,803 (12.8)
Northern Ireland	-	7,904 (21.1)
Scotland	-	1,850 (4.9)
Missing	15 (0.03)	8 (0.02)
**IMD ***		
1 (most deprived)	5,671 (12.2)	1,929 (12.8)
2	7,554 (16.2)	2,608 (17.4)
3	8,305 (17.9)	2,968 (19.8)
4	10,221 (22.0)	3,334 (22.2)
5 (least deprived)	14,780 (31.8)	4,189 (27.9)

Legend: SD (standard deviation), IMD (Index of Multiple Deprivation). * 22,401 (59.9%) of CPRD GOLD patients had missing data for IMD.
