# Supplementary material for: Exploring the impact of varying definitions of exacerbations of chronic obstructive pulmonary disease in routinely collected electronic medical records
Source: PLoS One. 2023 Nov 1;18(11):e0292876. doi: 10.1371/journal.pone.0292876 (PMC10619826; doi:10.1371/journal.pone.0292876)
Supplement: S2 Table — Legend: The proportion of frequent exacerbators each year of follow-up was determined for patients with active follow-up for each year of interest. Algorithm 1 was based off a validated algorithm using CPRD and HES, algorithm 2 included the validated CPRD algorithm but no HES, algorithm 3 included lower respiratory tract infection and prescribed medications for exacerbations, algorithm 4 included exacerbation of COPD codes in CPRD alone, algorithm 5 included oral corticosteroid prescriptions alone, and algorithm 6 included the validated CPRD and HES algorithm as well as accident and emergency HES data. (PDF) [file pone.0292876.s005.pdf]

Table S2: Proportion of frequent exacerbators for each year of patient follow-up by algorithm used to define exacerbations of COPD.

CPRD database	AECOPD definition	Year 1	Year 2	Year 3	Year 4	Year 5	Year 6	Year 7	Year 8	Year 9	Year 10
CPRD Aurum	Algorithm 1	42,189 (13.4)	37,682 (13.5)	34,165 (14.0)	28,554 (13.6)	23,937 (13.4)	20,175 (13.4)	17,209 (13.6)	14,262 (13.7)	11,584 (13.9)	9,353 (14.4)
	Algorithm 2	36,426 (11.6)	32,186 (11.5)	28,833 (11.8)	23,480 (11.2)	19,212 (10.7)	15,641 (10.4)	13,010 (10.3)	10,683 (10.3)	8,618 (10.3)	6,872 (10.6)
	Algorithm 3	20,146 (6.4)	18,341 (6.6)	16,547 (6.8)	13,781 (6.6)	11,603 (6.5)	9,614 (6.4)	7,935 (6.3)	6,569 (6.3)	5,337 (6.5)	4,327 (6.6)
	Algorithm 4	26,528 (8.4)	26,019 (9.3)	24,055 (9.9)	22,344 (10.6)	19,582 (11.0)	17,326 (11.5)	15,081 (12.0)	13,093 (12.6)	11,578 (13.9)	9,917 (15.2)
	Algorithm 5	15,570 (4.9)	14,842 (5.3)	13,669 (5.6)	10,810 (5.1)	8,148 (4.6)	5,831 (3.9)	4,313 (3.4)	2,954 (2.8)	1,934 (2.3)	1,229 (1.9)
	Algorithm 6	43,589 (13.8)	38,858 (13.9)	35,242 (14.4)	29,469 (14.0)	24,929 (13.9)	20,974 (13.9)	17,982 (14.3)	14,967 (14.4)	12,282 (14.7)	9,930 (15.2)
CPRD GOLD	Algorithm 1	43,922 (15.6)	39,162 (15.8)	36,357 (16.8)	30,185 (16.2)	25,648 (15.5)	20,750 (14.5)	16,349 (13.3)	13,048 (12.6)	10,230 (11.9)	8,039 (11.4)

Legend: The proportion of frequent exacerbators each year of follow-up was determined for patients with active follow-up for each year of interest. Algorithm 1 was based off a validated algorithm using CPRD and HES, algorithm 2 included the validated CPRD algorithm but no HES, algorithm 3 included lower respiratory tract infection and prescribed medications for exacerbations, algorithm 4 included exacerbation of COPD codes in CPRD alone, algorithm 5 included oral corticosteroid prescriptions alone, and algorithm 6 included the validated CPRD and HES algorithm as well as accident and emergency HES data.
